# Leveraging item-level accuracy and reaction time to address ceiling effects in the measurement of inhibitory control in preschool-aged children

**DOI:** 10.3389/fpsyg.2023.861441

**Published:** 2023-02-03

**Authors:** Michael T. Willoughby, Marie Camerota, Katherine Merseth King, Tabitha Nduku, Benjamin Piper

**Affiliations:** ^1^Education and Workforce Development, RTI International, Research Triangle Park, NC, United States; ^2^Department of Psychiatry and Human Behavior, Alpert Medical School of Brown University, Providence, RI, United States; ^3^International Education, RTI International, Washington, DC, United States; ^4^International Education, RTI International, Nairobi, Kenya

**Keywords:** early childhood, executive function, global south, lower middle income country, psychometrics

## Abstract

Preschool-aged children’s performance on inhibitory control tasks is typically represented by the overall accuracy of their item responses (e.g., mean proportion correct). However, in settings where children vary widely in age or ability level, inhibitory control tasks are susceptible to ceiling effects, which undermine measurement precision. We have previously demonstrated a general approach for scoring inhibitory control tasks that combines item-level accuracy and reaction-time information to minimize ceiling effects. Here, we extend that approach by incorporating additional item-level reaction time data from an adjunct (simple reaction time) task. We contrast three approaches for scoring inhibitory control tasks, two of which rely exclusively on item accuracy information and a third which also considers item reaction time information. We demonstrate the impacts of these different approaches to scoring with two inhibitory control tasks that were included in a recent evaluation of the Red Light, Purple Light intervention in preprimary classrooms in Nairobi County, Kenya. We limited our study to children who met inclusion criteria at pre-test (*N* = 418; 51% male; mean age = 4.8 years) or post-test (*N* = 386; 51% male; mean age = 4.8 years). Children’s performance on individual inhibitory control tasks was strongly correlated regardless of the scoring approach (*r*s = 0.73–0.97 across two tasks). However, the combined accuracy and reaction time scores eliminated ceiling effects that were common when only accuracy information was used. The combined accuracy and reaction time models also distinguished item-level RT into inhibitory control and processing speed components, which are distinct constructs. Results are discussed with respect to the challenges and nuances of the estimation and interpretation of inhibitory control task scores with children of varied ages and ability levels.

## 1. Introduction

Inhibitory control (IC) is a higher-order construct that subsumes a variety of cognitive and motivational processes that involve the suppression of a highly learned, prepotent, or appetitive responses. Numerous subdivisions of IC have been proposed, with Nigg’s (2000) taxonomy being the most expansive. Children’s growing capacity to exhibit IC is a cardinal feature of many prominent models of self-regulation (Kopp, 1982; Blair and Ursache, 2011; Mischel et al., 2011; Nigg, 2017; Bailey and Jones, 2019). Widespread interest in IC also derives from its association with multiple other domains of functioning, including specific aspects of psychological development (Carlson and Moses, 2001); academic achievement (Allan et al., 2014); risk for psychopathology (Lipszyc and Schachar, 2010); and adult health and financial outcomes (Moffitt et al., 2011).

Early demonstrations that performance-based tasks could be used to objectively measure IC in early childhood spurred widespread and longstanding interest in this approach (e.g., Gerstadt et al., 1994; Espy, 1997; Kochanska et al., 1997). All IC tasks that are used in early childhood characterize children’s IC ability as a function of the accuracy with which they respond to items that make prepotent demands. Despite the widespread use of this approach, many IC tasks are only useful for limited age ranges, before and after which floor and ceiling effects are common (Petersen et al., 2016). The limited age ranges during which performance-based tasks optimally measure IC complicates their use in longitudinal studies. Similar problems arise in cross-sectional studies that involve children of varied ages or ability levels. For example, researchers who study IC in preprimary and primary school settings in the Global South may be especially likely to encounter children of varied ages or ability levels due to widespread over-enrollment, which often reflects some combination of late school entry, lack of accessible pre-primary education, and unreported grade repetition (Crouch and Merseth, 2017). We encountered higher than expected ceiling effects for three IC tasks that were included in a recent evaluation of the Red Light, Purple Light (RLPL) classroom intervention used in preprimary classrooms in Nairobi County, Kenya (Willoughby et al., 2021). The primary motivation for the current study was to consider alternative ways to score IC tasks that are less susceptible to ceiling effects.

Whereas IC tasks that are used in early childhood are nearly always scored based on the accuracy with which items are completed, IC tasks that are used in middle childhood begin to be scored based on the speed at which items are completed. Specifically, for many IC tasks, older children answer all (or most) of the items correctly, and IC begins to be inferred from changes in the speed at which they answer target and non-target items (e.g., in the flanker task, differences in the speed at which children respond to a central item that is flanked by either congruent or incongruent items is used as an indication of IC). Although some have criticized a reliance on changes in RT within a task to index IC (Hedge et al., 2018; Draheim et al., 2019), this approach to scoring tasks is common. Notably, the criticisms of using RT difference scores to index IC are primarily psychometric in nature (owing to poor reliability of difference scores) and are not an indictment of the idea that individual differences in the speed of child responses are informative of individual differences in IC.

The NIH Toolbox flanker and dimensional change card sort tasks are two tasks that are increasingly used to measure IC (and, more broadly, executive function) in early childhood. Unlike most tasks that are used in early childhood, the NIH Toolbox tasks consider both the accuracy and speed of a child’s responses to generate continuous performance scores, using a “two-vector” approach to score tasks (Zelazo et al., 2013). Specifically, children’s overall accuracy and RT (i.e., median RT for incongruent items) are rescaled into 5-point scales. For children who answer <80% of test items correctly, their task scores are defined solely by their rescaled accuracy score (i.e., scores range from 0 to 5). For children who answer 80% or more of test items correctly, their task scores are defined by the summation of rescaled accuracy and RT metrics (i.e., scores range from 0 to 10). Although we appreciate the intent and intuitiveness of the two-vector scoring approach, including the mitigation of ceiling effects, this method suffers from at least five problems. Specifically, the two-vector approach: (1) imposes an arbitrary threshold for determining when accuracy information should be complemented by RT information; (2) presumes that accuracy and RT metrics are equally informative of IC for children who exceed the threshold; (3) presumes that standardization of accuracy and RT metrics is sufficient for placing them on a common scale; (4) presumes that accuracy and RT metrics are measured without error; and (5) presumes that RT metrics are solely indicative of IC (*cf.* general speed of processing). We are concerned that the widespread adoption of the NIH Toolbox for measuring IC in early childhood (and specifically the two-vector scoring approach, including the precedent that it sets for using this approach to score similar tasks) may yield inaccurate inferences and conclusions. Here, we introduce an alternative approach to scoring IC tasks that overcomes these limitations.

Psychometricians have long considered the role that RT data plays in measuring cognitive ability. This interest has grown with the increased use of computerized cognitive assessments (Kyllonen and Zu, 2016; De Boeck and Jeon, 2019). Molenaar and Visser (2017) distinguished two traditions for using RT data from cognitive assessments. In the psychometric tradition, RT is conceptualized as a source of interindividual differences in an underlying construct, which can be leveraged to improve the measurement precision of that construct. In the cognitive tradition, RT is conceptualized as a source of information about intraindividual differences in the strategies and processes that underlie task performance. Here, we consider a psychometric approach for addressing ceiling effects in IC tasks with preschool-aged children. That is, we did not go into this work with *a priori* ideas about the specific cognitive processes that children rely on when they encounter the IC tasks, and we do not assert that our analytic approach would support or refute specific cognitive models of task performance. We have a more pragmatic interest, which is focused on leveraging item-level accuracy and RT information to yield scores that are less susceptible to ceiling effects than traditional accuracy-only approaches to task scoring.

**Figure 1:**
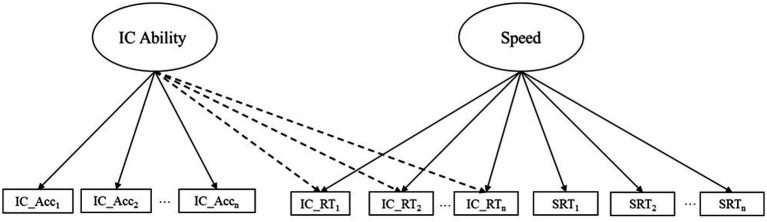
Exemplary two-factor confirmatory factor model for obtaining enriched scores. This model depicts the use of item-level accuracy and RT information from a generic inhibitory control task and item-level RT information from a simple reaction time task to create enriched inhibitory control scores. Dashed lines indicate cross-loadings. IC, inhibitory control; IC_Acc, accuracy from inhibitory control task; IC_RT, reaction time from inhibitory control task; SRT, reaction time from simple reaction time task.

A path diagram that depicts our general model of interest is presented in Figure 1. Three points are noteworthy. First, this model makes use of *item-level* information related to the accuracy and speed of child responses. Second, in contrast to our previous work that focused exclusively on item responses from IC tasks (Magnus et al., 2019; Camerota et al., 2020), we make joint use of item-level information from a simple RT task and a focal IC task. By incorporating information from the simple RT task into our models, we hope to better distinguish the speed of children’s item responses into general components of processing speed and IC ability. Third, our model is parameterized such that latent variables of processing speed and IC ability are uncorrelated, which is essential for decomposing item-level RT from the IC items into speed and ability components. This parameterization is inspired by a broader class of models that leverage item-level accuracy and RT information to improve the precision of cognitive ability measurement (see Molenaar et al., 2015). We applied this modeling approach to two IC tasks that were included in a previous intervention study and considered how these scores compared to traditional approaches that only consider item-level accuracy information.

## 2. Methods

### 2.1. Participants and procedures

This study involves the reanalysis of data that were included in a recent evaluation of the RLPL intervention that included 48 classrooms in 24 preprimary centers in Nairobi County, Kenya. Details on the sampling and randomization plan and the intervention content are detailed elsewhere (Willoughby et al., 2021). Briefly, 24 centers were randomized to RLPL or waitlist control conditions. Random assignment at the center level helped mitigate potential threats of contamination. Computerized (tablet) performance-based assessments of children’s executive function skills were individually administered at pre- and post-test assessments, which spanned ~8 weeks. This study was approved by the National Commission for Science, Technology, and Innovation, and by the Kenya Medical Research Institute.

In total, we collected data from 479 students at pretest and 438 students at post-test. For purposes of this study, we excluded children who were 7 years of age or older at the pretest assessment, who did not complete at least one of the two IC assessments that are our focus here (defined as responding to 70% of trials as described in *Measures*), or for whom there were questions about data quality (e.g., a few children appeared to have two assessments conducted at pre- or post-test). Assessors also occasionally switched the language of instruction during an assessment because of concerns about children’s task comprehension. In rare instances, assessors made different decisions about language across pretest and post-test. To improve data quality, we also excluded children who performed executive function tasks in more than one language so that EF task performance was not influenced by children’s listening comprehension skills. After these exclusions, we had usable data for 418 students at pretest (51% male; *Mage* = 4.8, *SD* = 0.8, *Range* = 3–6 years old; 61% assessments in Kiswahili) and 386 students at post-test (51% male; *Mage* = 4.8, *SD* = 0.7, *Range* = 3–6 years old; 60% assessments in Kiswahili).

### 2.2. Measures

Given time and cost constraints, our evaluation of the 8-week RLPL program was limited to student performance on tablet-based EF skills assessments that were previously validated for use in Kenya (Willoughby et al., 2019). The same tasks were administered at pre- and post-test assessments in the weeks immediately before and after the delivery of the RLPL intervention. All assessments were administered in preprimary centers. Although English is typically the language of instruction in urban Kenya, many children in Nairobi are more adept at Kiswahili. Consistent with our previous work, assessors determined the language of assessment during a rapport-building conversation with each child. Assessors began with a simple warm-up task that acclimated children to using a touch screen. Children subsequently completed a simple reaction-time task and five EF tasks. This study is limited to consideration of the simple reaction-time task and the two IC tasks that required children to touch one of two stimuli within a fixed interval of time. Both IC tasks followed a similar structure that involved the assessor reading a fully standardized script that included task instructions, a demonstration of how to complete sample items, and a presentation of training items to the child. Tasks were automatically discontinued if the child was unable to independently pass the training items after two attempts. We preprocessed item-level RT data. Specifically, following conventions in the literature (e.g., Wright and Diamond, 2014; Sulik and Obradovic, 2018), any item response that was recorded as being made faster than 400 milliseconds (ms) was considered implausible (i.e., responses at such a speed likely reflected a “trailing” response from the preceding item) and both the item-level accuracy and RT scores for that item were set to missing. In addition, all item-level RT data from the simple RT and IC tasks were log-transformed to reduce the influence of extreme values and to meet distributional assumptions. Because we present standardized parameter estimates, the interpretation of item-level RT is unchanged [i.e., higher values on log-transformed RT data index longer (slower) response times].

#### 2.2.1. Spatial conflict arrows

This 36-item spatial conflict task measured IC and cognitive flexibility. In this task, assessors instructed children to touch a button (on the right or left side of the screen) at which an arrow was pointing. In the first block, arrows appeared above the button at which they were pointing (spatially congruent block; 12 items). In the next block, the arrows appeared above the opposite button (spatially incongruent block; 12 items). In the final block, arrows appeared in a combination of previous locations (mixed block; 12 items). As described below, we created three scores for this task. Two scores made use of the item-level accuracy for 17 items (i.e., the 12 incongruent items from the incongruent block; five incongruent items from the mixed blocks). The third score made use of item-level accuracy for these same 17 items in addition to the item-level RT from 29 items (i.e., the 12 congruent items from the congruent block, the 12 incongruent items from the incongruent block; the five incongruent items from the mixed block). To minimize the influence of children who had substantial missing data, we limited our analyses to children who completed at least 70% of task items (i.e., children were included if at least 25 out of 36 items were non-missing). This criterion resulted in a total sample of *N* = 327 at pretest and *N* = 284 at post-test, with *N* = 215 contributing data at both timepoints.

#### 2.2.2. Silly sounds Stroop

This 17-item Stroop-like task measured IC. Each item presented pictures of a dog and a cat and the sound of either a dog barking or a cat meowing. The assessor instructed the child to touch the picture of the animal that did not make the sound (e.g., touching the cat when hearing a dog bark). As described below, we created three scores for this task. Two scores made use of the item-level accuracy from all 17 items. The third score made use of item-level accuracy for these same 17 items in addition to the item-level RT from these same 17 items. To minimize the influence of children who had substantial missing data, we limited our analyses to children who completed at least 70% of items on this task (i.e., children were included if at least 12 out of the 17 items on this task were non-missing). This criterion resulted in a total sample of *N* = 360 at pretest and *N* = 365 at post-test, with *N* = 303 contributing data at both timepoints.

#### 2.2.3. Bubbles

This 30-item task measured simple reaction time. A series of 30 bubbles of identical size, color, and shape appeared on the touch screen monitor, one at a time, and children were instructed to touch each bubble as fast as they could (successful touches resulted in a “popping” sound). Items were presented for up to 5,000 milliseconds (ms), and the time that transpired between stimuli onset and the child’s touch of the bubble was recorded. If an item was not touched, the item was considered inaccurate and the reaction time (RT) for that item was not recorded. Consistent with IC tasks, item responses that were faster than 400 ms were considered too fast to be plausible and were set to missing. Item-level RT was used in measurement models, and the mean RT across all valid items was used to index simple reaction time in descriptive analyses.

### 2.3. Analysis plan

We used three different approaches to score IC tasks at pretest and posttest assessments. First, we constructed a mean accuracy score for each task (i.e., the proportion of items on each task that made inhibitory demands that were answered correctly). These traditional task scores represent the typical approach to scoring IC tasks in early childhood. Second, we fit a one-factor confirmatory factor model (CFA) to the same items and used factor score estimation to obtain a task score. Whereas mean accuracy scores assume that all items are interchangeable indicators of IC ability, accuracy-only factor scores allow items to make differential contributions to IC ability. Third, we fit a two-factor CFA model to item-level accuracy and RT information from each IC task, as well as the item-level RT information from the Bubbles simple reaction time task. This model conformed to Figure 1 and was of primary interest. We used factor score estimation to obtain IC ability and processing speed scores from this model. We refer to the factor score estimates of the IC ability factor as enriched scores because they incorporated information about the accuracy and speed of children’s responses. Notably, a subset of RT items from each IC task cross-load on the ability and processing speed factors. Because these factors are orthogonal, the model decomposes this item-level RT information into ability and processing speed components (all the remaining accuracy and RT items load exclusively on the ability or processing speed factors). After both IC tasks were scored using all three methods, we presented within- and across-task comparisons, with a special interest in the extent to which the enriched scores overcome problems with ceiling effects that are evident for scores than only use accuracy information. Consistent with our previous analysis of these data (Willoughby et al., 2021), we used mixed linear models to test whether there were treatment effects using these enriched factor scores (i.e., we regressed each posttest score on the corresponding pretest score and a dichotomous indicator of treatment condition).

All measurement models were implemented using *Mplus* v8.6 (Muthén and Muthén, 1998-2020) and used a robust full information maximum likelihood estimator with numerical integration. As a result of using numerical integration, model fit statistics were not available. An exemplary *Mplus* script that corresponds to Figure 1 is provided in Supplementary material. Mixed linear models were implemented in SAS^®^ v9.

## 3. Results

### 3.1. Descriptive statistics

Descriptive statistics for the simple RT task and traditional scores for the IC tasks are displayed in Table 1. Overall, children performed well on both IC tasks at pretest and posttest (children answered 58% and 65% of incongruent items on the Arrows task correctly and 84% and 88% of Silly Sounds Stroop items correctly at pre- and posttest, respectively). Ceiling effects were common at pre- and (especially) post-test assessments (21%–41% of children answered all items correctly on each IC task). Simple RT was modestly inversely correlated with IC task scores at both pretest (*r* = −0.14, *p* < 0.02) and post-test (*r* = −0.20 to −0.22, *p* < 0.001); children who responded more accurately on the IC tasks also answered items on the simple RT task more quickly.

**Table 1:** Descriptive statistics and correlations among observed mean scores for inhibitory control tasks.

	1.	2.	3.	4.	5.	6.
1. Spatial conflict arrows (Pretest)	–					
2. Spatial conflict arrows (Posttest)	0.46***	–				
3. Silly sounds (Pretest)	0.21***	0.16*	–			
4. Silly sounds (Posttest)	0.16**	0.33***	0.45***	–		
5. Simple reaction time (Pretest)	−0.14*	−0.14*	−0.14**	−0.15*	–	
6. Simple reaction time (Posttest)	−0.10	−0.20**	−0.20***	−0.22***	0.52***	–
*N*	327	284	360	365	378	372
*M*	0.58	0.65	0.84	0.88	7.02	6.93
SD	0.38	0.38	0.21	0.18	0.19	0.16
Ceiling %	21%	30%	33%	41%	–	–

Ns = 215–364; +*p* < 0.10, *p < 0.05, **p < 0.01, ***p < 0.001. These scores represent children’s average performance on the subset of items from each task that make inhibitory control demands, as described in the Measures section. Ceiling % represents % of children who obtained the maximum task score. Simple reaction time (in seconds) has been log transformed.

### 3.2. Model fitting

As described in the Analysis Plan, we estimated one-factor (ability inferred from item accuracy) and two-factor (ability inferred from item accuracy and RT) CFA models for both IC tasks, separately at the pretest and posttest assessments (i.e., 2 models × 2 tasks × 2 measurement occasions = 8 models in total). Given the central importance of the two-factor models, which generated the enriched scores, we focus on the final parameter estimates from these models here (the final parameter estimates for the one-factor CFA models are presented in Supplementary material).

#### 3.2.1. Arrows task

The Arrows task included 17 items that were indicative of IC ability. In the two-factor, enriched CFA model at pretest, there were significant, positive loadings of accuracy (*λ* = 0.71–0.96, *p* < 0.001) on IC ability. Of 17 task RT items, 13 had significant, positive loadings (*λ* = 0.25–0.47, *p* < 0.05) on IC ability. There were significant, negative loadings of 14 of 17 incongruent RT (*λ* = −0.49 to −0.23, *p* < 0.02), 10 of 12 congruent RT (*λ* = −0.33 to −0.19, *p* < 0.003), and 30 simple RT (*λ* = −0.77 to −0.23, *p* < 0.001) indicators on latent speed (see Table 2). Overall, this indicated that more accurate and slower (larger RT) responses were indicative of better IC ability, while faster (smaller RT) responding was indicative of greater speed. As expected, accuracy items were more strongly indicative of IC ability than item RT, whereas simple RT was the strongest indicator of speed. Similar findings were observed at posttest (see Table 2).

**Table 2:** Final Standardized loadings from the enriched arrows task.

	Pretest			Posttest		
	Latent ability		Latent speed˟	Latent ability		Latent speed˟
Item	*λ* (Acc)	*λ* (RT)	*λ* (RT)	*λ* (Acc)	*λ* (RT)	*λ* (RT)
Arrows1	–	–	−0.20	–	–	−0.24
Arrows2	–	–	−0.19	–	–	−0.34
Arrows3	–	–	−0.27	–	–	−0.39
Arrows4	–	–	−0.28	–	–	−0.31
Arrows5	–	–	−0.05^+^	–	–	−0.25
Arrows6	–	–	−0.19	–	–	−0.17
Arrows7	–	–	−0.25	–	–	−0.33
Arrows8	–	–	−0.33	–	–	−0.33
Arrows9	–	–	−0.10^+^	–	–	−0.11^+^
Arrows10	–	–	−0.21	–	–	−0.26
Arrows11	–	–	−0.30	–	–	−0.34
Arrows12	–	–	−0.35	–	–	−0.28
Arrows13	0.73	0.35	−0.38	0.70	0.30	−0.32
Arrows14	0.90	0.34	−0.25	0.85	0.21	−0.36
Arrows15	0.91	0.41	−0.38	0.91	0.24	−0.39
Arrows16	0.89	0.47	−0.38	0.93	0.44	−0.43
Arrows17	0.71	0.28	−0.13^+^	0.86	0.31	−0.18
Arrows18	0.88	0.25	−0.32	0.92	0.26^+^	−0.25
Arrows19	0.92	0.32	−0.23	0.96	0.53	−0.26
Arrows20	0.85	0.34	−0.29	0.96	0.29	−0.21
Arrows21	0.85	0.41	−0.09^+^	0.90	0.51	−0.20
Arrows22	0.96	0.26^+^	−0.31	0.91	0.35	−0.21
Arrows23	0.96	0.44	−0.44	0.96	0.34	−0.30
Arrows24	0.93	0.32	−0.41	0.96	0.50	−0.22
Arrows27	0.84	0.17^+^	−0.39	0.82	0.38	−0.27
Arrows28	0.86	0.19^+^	−0.33	0.92	0.36	−0.27
Arrows31	0.81	0.35	−0.32	0.82	0.40	−0.33
Arrows32	0.90	0.30	−0.49	0.88	0.44	−0.28
Arrows33	0.73	0.25^+^	−0.15^+^	0.85	0.31	−0.12^+^

*N* = 327 and 284 for pretest and posttest models, respectively. Arrows items 1–12, 13–24, and 25–36 are the congruent, incongruent, and mixed blocks, respectively. All factor loadings in table are significant at *p* < 0.05 except where marked (^+^). The congruent items in the mixed block (i.e., Arrows items 25, 26, 29, 30, 34, 35, and 36) were not used as indicators of either latent ability or speed.

˟To conserve space, we do not present parameter estimates for the 30 items from the Bubbles task that were also included as indicators of latent speed (pretest standardized *λ* = −0.77 to −0.23, posttest standardized *λ* = −0.77 to −0.35 all *p*s < 0.001).

#### 3.2.2. Silly sounds Stroop task

The Silly Sounds Stroop task included 17 items that were indicative of IC ability. In the two-factor, enriched CFA model at pretest, there were significant, positive loadings of accuracy on IC ability (*λ* = 0.38–0.84, *p* < 0.001). There were significant, negative loadings for 14 of the 17 task RT items on IC ability (*λ* = −0.52 to −0.23, *p* < 0.05). Unlike in the Arrows model, where slower responses were indicative of better IC ability, these results indicate that more accurate and *faster* responding on the Silly Sounds Stroop Task is indicative of better IC ability. Like in the Arrows model, there were significant, negative loadings for 11 of the 17 task RT (*λ* = −0.12 to −0.31, *p* < 0.03) and 30 simple RT (*λ* = −0.78 to −0.33, *p* < 0.001) indicators on latent speed (see Table 3). Similar findings were observed at posttest (see Table 3), although all 17 task RT items loaded significantly and negatively on IC ability at posttest (*λ* = −0.54 to −0.22, *p* < 0.02).

**Table 3:** Final standardized loadings from the enriched silly sounds stroop task.

	Pretest			Posttest		
	Latent ability		Latent speed˟	Latent ability		Latent speed˟
Item	*λ* (Acc)	*λ* (RT)	*λ* (RT)	*λ* (Acc)	*λ* (RT)	*λ* (RT)
Silly1	0.49	−0.39	−0.26	0.40	−0.29	−0.19
Silly2	0.62	−0.39	−0.23	0.37	−0.44	−0.22
Silly3	0.74	−0.44	−0.25	0.59	−0.51	−0.22
Silly4	0.62	−0.33	−0.31	0.69	−0.27	−0.19
Silly5	0.84	−0.51	−0.20	0.89	−0.54	−0.21
Silly6	0.83	−0.52	−0.23	0.81	−0.54	−0.19
Silly7	0.52	−0.48	−0.15	0.53	−0.53	−0.21
Silly8	0.60	−0.16^+^	−0.07^+^	0.57	−0.27	−0.19
Silly9	0.51	−0.23	−0.07^+^	0.55	−0.22	−0.13
Silly10	0.60	−0.32	−0.08^+^	0.49	−0.35	−0.25
Silly11	0.56	−0.43	−0.12	0.36	−0.40	−0.17
Silly12	0.63	−0.17^+^	−0.13^+^	0.88	−0.30	−0.20
Silly13	0.38	−0.42	−0.15	0.47	−0.29	−0.20
Silly14	0.73	−0.45	−0.22	0.71	−0.29	−0.16
Silly15	0.73	−0.38	−0.08^+^	0.67	−0.40	−0.16
Silly16	0.61	−0.34	−0.03^+^	0.68	−0.42	−0.22
Silly17	0.57	−0.20^+^	−0.18	0.48	−0.27	−0.19

*N* = 360 and 365 for pretest and posttest models, respectively. All factor loadings in table are significant at *p* < 0.05 except where marked (^+^). ˟To conserve space, we do not present parameter estimates for the 30 items from the Bubbles task that were also included as indicators of latent speed (pretest standardized *λ* = −0.78 to −0.23, posttest standardized *λ* = −0.78 to −0.33 all *p*s < 0.001).

### 3.3. Score comparisons

**Figure 2:**
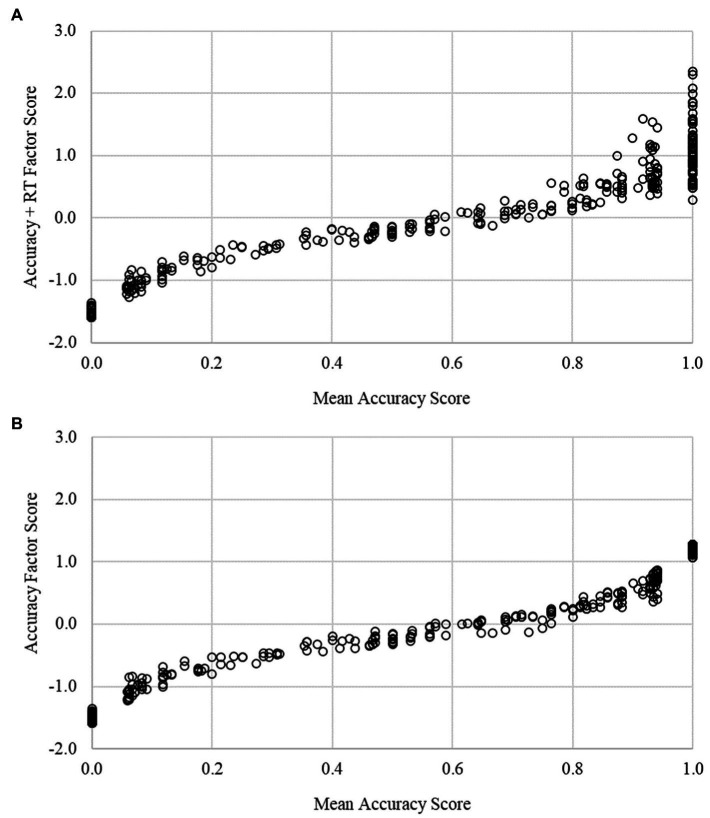
Score comparisons for the Arrows task. This figure plots mean accuracy scores against the enriched (**A**) and accuracy (**B**) factor scores. Ceiling effects (mean accuracy scores equal to 1.0) are mitigated using enriched, but not accuracy, factor scores.

**Figure 3:**
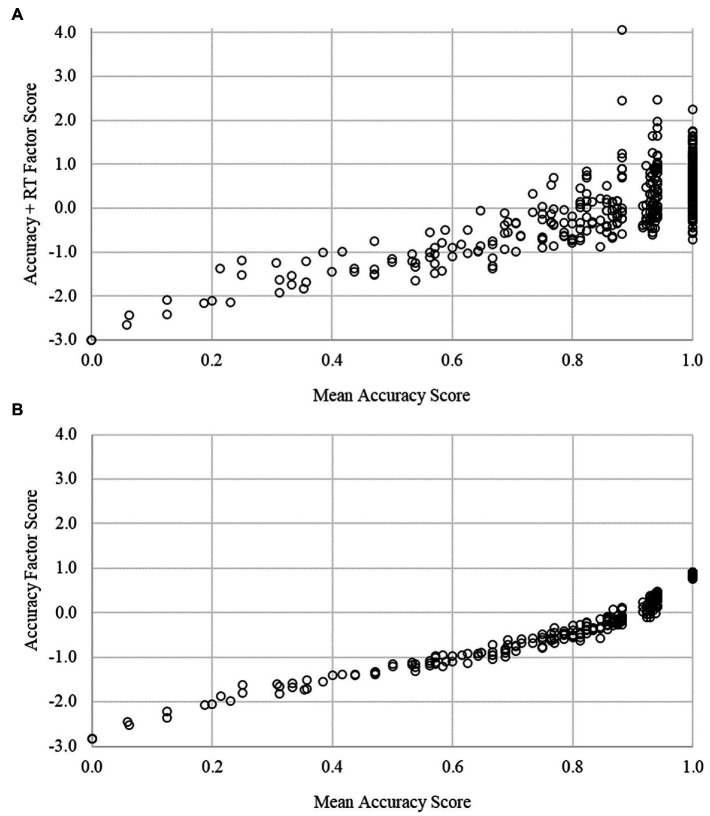
Score comparisons for the Silly Sounds Stroop task. This figure plots mean accuracy scores against the enriched (**A**) and accuracy (**B**) factor scores. Ceiling and near ceiling effects (mean accuracy scores >0.8) are mitigated using enriched, but not accuracy, factor scores.

We compared traditional scores (mean percent correct), accuracy-only factor scores, and enriched (accuracy + RT) factor scores for each IC task at the pretest and posttest assessments (Table 4). Traditional, accuracy-only, and enriched scores were strongly correlated for the Arrows (*r* = 0.91–0.97, *p*s < 0.001) and Silly Sounds Stroop (*r* = 0.73–0.95, *p* < 0.001) tasks. Despite the high degree of rank order stability in scores, a visual characterization of score overlap revealed a more nuanced set of results. Figures 2A, 3A depict the association between the enriched factor and traditional (mean accuracy) scores. For both Arrows and Silly Sounds Stroop tasks, a wide range of factor scores existed for children who answered all items accurately (i.e., those scoring at ceiling). Hence, although the rank order stability of score performance is largely unchanged for children in the middle of the score distribution, the enriched scores provide improved precision of measurement for children who answered all items correctly. Figures 2B, 3B depict the association between the accuracy-only factor and traditional (mean accuracy) scores. These results demonstrate it is the incorporation of RT items, not the differential weighting of accuracy items, that overcomes problems with ceiling effects.

**Table 4:** Bivariate correlations between inhibitory control task scores.

Task (Score)	1.	2.	3.	4.	5.	6.
1. Arrows (Mean)	0.46***	0.96***	0.91***	0.33***	0.33***	0.25***
2. Arrows (Accuracy)	0.97***	0.45***	0.94***	0.31***	0.31***	0.23***
3. Arrows (Enriched)	0.95***	0.96***	0.40***	0.31***	0.32***	0.18**
4. Silly Sounds (Mean)	0.21***	0.21***	0.20**	0.45***	0.94***	0.74***
5. Silly Sounds (Accuracy)	0.22***	0.22***	0.21***	0.95***	0.42***	0.73***
6. Silly Sounds (Enriched)	0.24***	0.23***	0.18**	0.78***	0.77***	0.57***

Ns = 269–360 for pretest and Ns = 263–365 for posttest; *p < 0.05, **p < 0.01, ***p < 0.001; values below and above the diagonal are from the pretest and posttest assessments, respectively; values on the diagonal (bolded) represent pre-post stability; Mean –proportion of inhibitory control items answered correctly; Accuracy – factor score estimate of ability using task accuracy items; Enriched – factor score estimate of ability using task accuracy and a combination of task and Bubbles RT items.

The diagonal of Table 4 summarizes the stability of children’s performance on IC tasks between the pre- and post-test assessments. The stability of children’s performance on the Arrows task was comparable for all scoring methods (*r*s = 0.40–0.46). The stability of children’s performance on the Silly Sounds Stroop task was modestly stronger for enriched (*r* = 0.57) relative to traditional and accuracy-only factor scores (*r*s = 0.42, 0.45).

### 3.4. Treatment effects

We examined the impact of the RLPL intervention on IC ability using the enriched factor scores. As in our previous work, we found no evidence of an RLPL intervention effect on IC ability for either IC task (*p*s > 0.07). Effect sizes were similar for traditional and enriched scores for Arrows (*Cohen’s d* = −0.08 vs. −0.10, respectively) and Silly Sounds Stroop (*Cohen’s d* = −0.14 vs. −0.11, respectively) tasks.

## 4. Discussion

Most of what is known about executive function skills, generally, and IC skills, specifically, is based on research that was conducted in high-income countries; however, these skills are also germane to early learning and school readiness in the Global South (Obradovic and Willoughby, 2019). Regardless of where IC tasks are used, the presence of children of varied ages and abilities complicates the selection and interpretation of IC tasks, which often exhibit floor and especially ceiling effects.

IC tasks that are used with young children nearly always have a speeded component. That is, children are instructed to make fast responses to stimuli that appear for a short, fixed duration of time. Although young children’s performances on IC tasks are typically inferred from the accuracy of their responses, as children get older, the speed of their responses is understood to reflect individual differences in their IC and general speed of processing (Davidson et al., 2006; Willoughby et al., 2018). Here, we demonstrated an approach for scoring IC tasks in early childhood that makes joint use of the accuracy and speed of children’s task performance. We believe that our approach overcomes the aforementioned limitations of the two-vector approach that is used to score EF tasks in the NIH Toolbox. Specifically, our approach uses item-level information about accuracy and RT information, does not require an arbitrary threshold for determining whether RT information should contribute to task scores, does not assume that accuracy and RT contribute equally to scores, acknowledges measurement error in accuracy and RT information, and acknowledges that item-level RT information can convey information about both speed of processing and IC ability. Notably, our enriched IC task scores were strongly correlated with traditional task scores. However, these strong correlations obscured the fact that the enriched scores help to distinguish performance between children who performed extremely well on each task, thereby mitigating ceiling effects. Attending to ceiling effects in these data were the primary impetus for this work. Figures 2, 3 help to delineate the primary contribution of our enriched scores relative to traditional scores. Moreover, they demonstrate that it is the inclusion of RT information, not simply the differential weighting of accuracy items, that mitigates ceiling effects.

It is noteworthy that the speed at which children responded to items had a differential impact on their IC skills across tasks. Whereas *faster responses* were associated with improved ability in the Silly Sound Stroop task, *slower responses* were associated with improved ability in the Arrows task. We suspect that these differences reflect task demands. In the Silly Sound Stroop task, children had a consistent and relatively simple decision to make (e.g., touch the dog picture every time that you hear the “meow” sound). In the Arrows task, children’s decisions about which button to touch were informed by the orientation of the stimulus (e.g., touch the left button on the screen for left-pointing arrows); however, the varied spatial location of the stimulus (e.g., sometimes left-pointing arrows appeared on the left side and at other times on the right side) complicated decision-making. Slower RT in the Arrows task may reflect a more deliberate approach to task completion (decisions are conditional on spatial location of stimulus). Taken together, these results demonstrate the generality of our analytic approach and underscore the varied ways in which item-level RT may inform IC ability in young children. Although the incorporation of item-level RT helped to address ceiling effects, the enriched scores exhibited a similar cross-time stability as the traditional scores. Moreover, there was no indication that the magnitude of treatment effects differed for enriched vs. traditional scores.

In this study, we focused on two IC tasks (i.e., Arrows and Silly Sounds Stroop tasks) that included item-level information regarding the accuracy and speed at which children respond to incongruent items. In the parent study, we administered a third IC task (i.e., Animal Go/No-Go task), which was not considered here. In go/no-go paradigms, children are instructed to withhold a response to no-go trials, following a sequence of go trails (e.g., touch every animal as fast as you can, unless it is a pig). In the Animal Go/No-Go task, we only had information about the speed at which children made *incorrect* responses to no-go items (i.e., correct responses to no-go items involve abstaining from making a response, such that item-level RT is structurally missing). In analyses that were not presented here, we demonstrated that item-level RT information in the Animal Go/No-Go tasks did not help address ceiling effects. Unsurprisingly, knowing how quickly a child answers a no-go item incorrectly does not help to address ceiling effects. It is an open question as to whether the analytic approach that we demonstrated here may be useful for no-go tasks in situations where floor effects are common.

Our study is characterized by at least three limitations. First, we estimated a relatively large number of parameters with a relatively modest sized sample. Although we did not encounter any difficulties with maximum likelihood estimation, alternative estimators may be warranted in situations with many items and small sample sizes. Relatedly, although we had pre and posttest data available, we estimated models separately at each measurement occasion. Future studies with larger samples and with repeated measures that span longer periods of time will be in a better position to consider tests of longitudinal measurement invariance for combined accuracy and RT models. Second, we applied the same psychometric model to both IC tasks, without consideration of whether the cognitive processes that underlie these tasks varied. While we consider the generality of our approach a strength, others may consider this approach a limitation (favoring “cognitive” vs. “psychometric” models of IC tasks). Third, we excluded a relatively small number of children from this study who were ≥ 7 years old and enrolled in preprimary classrooms. It is an open question about how broad of an age span or skill level our enriched approach to task scoring can accommodate. For example, we recently encountered an instance where the contribution of item-level RT to IC ability on the same task varied across children of different ability levels despite similarity in ages (see Camerota et al., 2020). Specifically, among children who performed comparatively poorly on an IC task, slower RT was indicative of better IC; conversely, among children who performed comparatively better on an IC task, faster RT was indicative of better IC. Differences in the contributions that RT makes to task performance may be indicative of developmental changes in the strategies that children use when encountering a task (see, e.g., Chevalier et al., 2013). To the extent that this is true, this complicates any approach to task scoring.

The primary contribution of this study is to demonstrate an approach for using item-level accuracy and RT data to score IC tasks. Although this approach to scoring tasks is admittedly more complicated than traditional approaches, the extra effort may be warranted in situations where ceiling effects are expected due to the inclusion of children of mixed ages or skill levels. We have provided an exemplary *Mplus* script in Supplementary material section to facilitate other researchers’ consideration of this approach. This script will be understood by individuals who have some familiarity with generalized structural equation modeling and is intended to be generic (it does not correspond to our tasks). We hope that this study will spur more widespread interest in (and criticism of) efforts to improve the measurement of IC skills in early childhood. Our approach can be easily extended in important ways. For example, to the extent that there are child-level characteristics (e.g., history of HIV exposure; low birth weight) that may influence either IC or processing speed, incorporating these variables as predictors of the latent variables of inhibitory control or speed of processing may further improve the precision of IC measurement in ways that we did not consider here (see, e.g., Curran et al., 2016, 2018). Given the substantial time and money that goes into collecting performance-based measures of IC with young children, we believe that additional efforts to improve the resulting task scores are worth the added effort.

## Data availability statement

The datasets presented in this article are not readily available because we did not explicitly include this language in our consent forms. Requests to access the datasets should be directed to MW, mwilloughby@rti.org.

## Ethics statement

The studies involving human participants were reviewed and approved by National Commission for Science, Technology, and Innovation, and by the Kenya Medical Research Institute. Written informed consent to participate in this study was provided by the participants’ legal guardian/next of kin.

## Author contributions

MW contributed to study design, conceived of the manuscript, and took primary responsibility for manuscript writing. MC conducted statistical analyses and assisted with manuscript writing. KK, TN, and BP contributed to the conceptualization and execution of the parent study and assisted with manuscript editing. All authors contributed to the article and approved the submitted version.

## Funding

This study was conducted using internal funding from RTI International.

## Conflict of interest

The authors declare that the research was conducted in the absence of any commercial or financial relationships that could be construed as a potential conflict of interest.

## Publisher’s note

All claims expressed in this article are solely those of the authors and do not necessarily represent those of their affiliated organizations, or those of the publisher, the editors and the reviewers. Any product that may be evaluated in this article, or claim that may be made by its manufacturer, is not guaranteed or endorsed by the publisher.
