# Supplementary material for: Leveraging item-level accuracy and reaction time to address ceiling effects in the measurement of inhibitory control in preschool-aged children
Source: Front Psychol. 2023 Feb 3;14:861441. doi: 10.3389/fpsyg.2023.861441 (PMC9937058; doi:10.3389/fpsyg.2023.861441)
Supplement: Supplementary file 1 [file Data_Sheet_1.pdf]

Supplementary Exhibit. Mplus Script for Enriched Factor Score of a Generic 10-Item Inhibitory Control and 30-Item Simple Reaction Time Task.

```
Title: Exemplary Script for Enriched Scoring of Inhibitory Control Task
!Assumes 10 item IC task in which acc & RT are all relevant;
!Assumes 30 item SRT task;

Data: File is IC.dat;

Variable:
Names are id_num IC_ACC1- IC_ACC10
                 IC_RT1- IC_RT10
                 SRT1-SRT30;

Usevariables= IC_ACC1- IC_ACC10 IC_RT1- IC_RT10 SRT1-SRT30;
Categorical= IC_ACC1- IC_ACC10;

Auxiliary=id_num;

Missing are .;

Analysis:
 Estimator=MLR;
 Type=complex;

 Model:

IC_ABILITY by IC_ACC1- IC_ACC10*;        !Loading of task accuracy on latent IC;
IC_ABILITY by IC_RT1- IC_RT10*;          !Crossloading of task RT on latent IC;

SPEED by IC_RT1- IC_RT10*-.2;            !Loading of task RT on latent speed;
SPEED by SRT1-SRT30*-.5 ;                !Loading of simple RT on latent speed;

IC_ABILITY with SPEED@0;                 !IC and speed are uncorrelated;

[IC_ACC1$1-IC_ACC10$1];                  !Thresholds for task accuracy;
[IC_RT1-IC_RT10];                        !Intercepts for task RT;
[SRT1-SRT30];                            !Intercepts for simple RT;

IC_ABILITY @1; [IC_ABILITY @0];          !Set scale of latent IC;
SPEED@1; [SPEED@0];                      !Set scale of latent speed;

Output: stdyx sampstat;

Savedata:                                !Save enriched factor scores;
file is FScores.txt;
save is fscores;
```

Supplemental Table 1. Standardized Loadings of Accuracy on Ability from One-Factor Scoring Models (Arrows Task)

	Pretest	Posttest
Item	λ (Acc)	λ (Acc)
Arrows1	--	--
Arrows2	--	--
Arrows3	--	--
Arrows4	--	--
Arrows5	--	--
Arrows6	--	--
Arrows7	--	--
Arrows8	--	--
Arrows9	--	--
Arrows10	--	--
Arrows11	--	--
Arrows12	--	--
Arrows13	0.75	0.70
Arrows14	0.91	0.86
Arrows15	0.92	0.91
Arrows16	0.90	0.93
Arrows17	0.71	0.87
Arrows18	0.88	0.92
Arrows19	0.91	0.96
Arrows20	0.86	0.96
Arrows21	0.86	0.91
Arrows22	0.97	0.93
Arrows23	0.97	0.97
Arrows24	0.95	0.96
Arrows27	0.85	0.82
Arrows28	0.88	0.92
Arrows31	0.81	0.81
Arrows32	0.90	0.89
Arrows33	0.77	0.84

Note: N = 327 and 284 for pretest and posttest models, respectively. Arrows items 1-12, 13-24, and 25 – 36 are the congruent, incongruent, and mixed blocks, respectively. All factor loadings in table are significant at $p < .001$. The congruent items in the mixed block (i.e., Arrows items 25, 26, 29, 30, 34, 35, and 36) were not used as indicators of either latent ability or speed.

Supplemental Table 2. Standardized Loadings of Accuracy on Ability from One-Factor Scoring Models (Silly Sounds Stroop Task)

	Pretest	Posttest
Item	λ (Acc)	λ (Acc)
Silly1	0.57	0.56
Silly2	0.58	0.55
Silly3	0.75	0.70
Silly4	0.74	0.82
Silly5	0.85	0.92
Silly6	0.81	0.88
Silly7	0.52	0.78
Silly8	0.69	0.67
Silly9	0.63	0.72
Silly10	0.69	0.58
Silly11	0.73	0.53
Silly12	0.68	0.87
Silly13	0.59	0.56
Silly14	0.81	0.76
Silly15	0.79	0.75
Silly16	0.76	0.72
Silly17	0.64	0.70

Note: N = 360 and 365 for pretest and posttest models, respectively. All factor loadings in table are significant at $p < .001$.
